# Eco-Friendly Textile-Based Wearable Humidity Sensor with Multinode Wireless Connectivity for Healthcare Applications

**DOI:** 10.1021/acsabm.4c00593

**Published:** 2024-07-04

**Authors:** Ajay Beniwal, Gaurav Khandelwal, Rudra Mukherjee, Daniel M. Mulvihill, Chong Li

**Affiliations:** James Watt School of Engineering, University of Glasgow, Glasgow G12 8QQ, U.K.

**Keywords:** humidity sensor, textile, eco-friendly, wearable sensor, PEDOT:PSS, healthcare applications

## Abstract

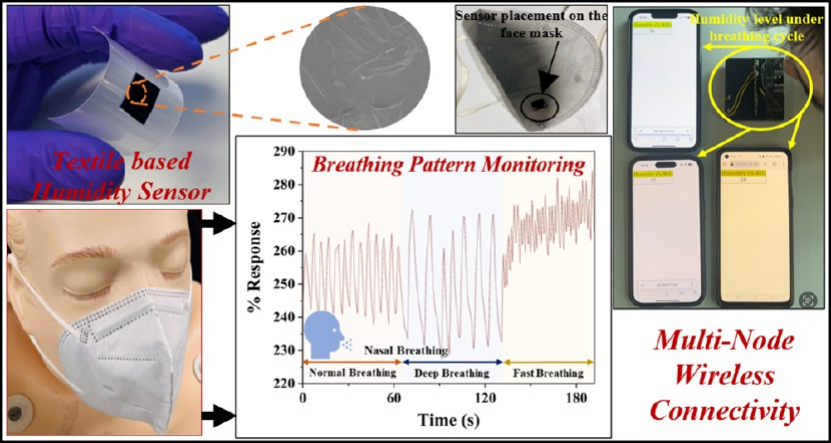

Textile-based wearable humidity sensors are of great interest for human healthcare monitoring as they can provide critical human-physiology information. The demand for wearable and sustainable sensing technology has significantly promoted the development of eco-friendly sensing solutions for potential real-world applications. Herein, a biodegradable cotton (textile)-based wearable humidity sensor has been developed using fabsil-treated cotton fabric coated with a poly(3,4-ethylenedioxythiophene):poly(styrenesulfonate) (PEDOT:PSS) sensing layer. The structural, chemical composition, hygroscopicity, and morphological properties are examined using X-ray diffraction (XRD), Fourier transform infrared spectroscopy (FTIR), contact angle measurement, and scanning electron microscopy (SEM) analysis. The developed sensor exhibited a nearly linear response (Adj. *R*-square value observed as 0.95035) over a broad relative humidity (RH) range from 25 to 91.5%RH displaying high sensitivity (26.1%/%RH). The sensor shows excellent reproducibility (on replica sensors with a margin of error ±1.98%) and appreciable stability/aging with time (>4.5 months), high flexibility (studied at bending angles 30°, 70°, 120°, and 150°), substantial response/recovery durations (suitable for multiple applications), and highly repeatable (multicyclic analysis) sensing performance. The prospective relevance of the developed humidity sensor toward healthcare applications is demonstrated via breathing rate monitoring (via a sensor attached to a face mask), distinguishing different breathing patterns (normal, deep, and fast), skin moisture monitoring, and neonatal care (diaper wetting). The multinode wireless connectivity is demonstrated using a Raspberry Pi Pico-based system for demonstrating the potential applicability of the developed sensor as a real-time humidity monitoring system for the healthcare sector. Further, the biodegradability analysis of the used textile is evaluated using the soil burial degradation test. The work suggests the potential applicability of the developed flexible and eco-friendly humidity sensor in wearable healthcare devices and other humidity sensing applications.

## 1 Introduction

Humidity sensors have drawn significant attention from the scientific community owing to their applicability in multiple application areas including the healthcare sector, agricultural science, environmental control, and various biomedical processes.^1−5^ Humidity sensors based on different transduction mechanisms, such as resistance, capacitance, field effect transistors (FETs), and optical fiber have been long established.^6−13^ Among them, resistive humidity sensors are the most attractive thanks to their low fabrication cost, easy device integration and signal acquisition, cost-effectiveness, easy manufacturing, and low power consumption.^1,11,14^ Further, humidity sensors based on flexible and wearable electronics can play an important role, especially for personal and wearable healthcare applications.^15,16^ To attain favorable flexibility, miscellaneous ductile materials such as poly(ethylene terephthalate) (PET), poly(dimethylsiloxane) (PDMS), paper, and poly(ethylene naphthalate) (PEN) have been extensively explored and utilized to develop wearable humidity sensors.^8,17−20^ However, limited breathability and hygroscopicity properties of polymer film-based sensors significantly lower their comfort and sensitivity; whereas, vulnerability and wetness wrinkles are the major shortcomings of paper-based sensors.^21^ Recently, textile (cotton) has been explored as an appropriate substitute for the development of wearable electronic sensing devices.^22−25^ Their remarkable properties like wearability, excellent flexibility, knittability, superior mechanical compliance, and conformability make them extremely suitable for wearable sensing technologies.^8,13,20^ Further, owing to their structural, hygroscopic, breathable, and biodegradable properties, the textiles seem highly suitable for developing humidity sensors along with promoting a shift toward eco-friendly electronics.^21,26^

Moreover, the need for humidity sensors is absolutely necessary as deviations in air humidity from the preferred levels could critically impact on quality of life because the human body feels most comfortable when the surrounding humidity is around/within the ∼40–70%RH range.^11,20^ Along with this, humidity sensors have the potential to be effectively utilized for human breath monitoring as abnormal breathing or respiration rate could be an indication of a physical problem^27^ and also related to several health conditions like bronchitis, heart diseases, pneumonia, chronic obstructive pulmonary disease (COPD), sleep apnea syndrome (SAS), asthma, etc.^27−30^ The exhaled human breath usually contains relative humidity within/around the range of ∼41.9–91.0%RH.^31^ Also, humidity sensors have potential applicability to other healthcare applications including skin moisture monitoring, neonatal care, electronic skin, etc.^32−35^ These applications demand a highly sensitive humidity sensor having real-time stable response within the desired humidity range.

In this work, a cotton (textile)-based eco-friendly and resistive-type humidity sensor is developed by using PEDOT:PSS as the active layer material to enable a wide humidity sensing range from 25 to 91.5%RH, which is highly suitable for healthcare applications and environmental monitoring. The choice of PEDOT:PSS as the sensing material is inspired by its eco-friendly and biocompatible properties, ease of process, good thermal stability, and appropriateness toward solution-based techniques.^36,37^ Prior to PEDOT:PSS coating, the hydrophobicity of the substrate is enhanced via fabsil treatment to achieve stable sensing response and make it suitable for humidity sensing applications. The important sensing parameters such as % response, response and recovery times, repeatability, reproducibility, and stability are systematically analyzed and discussed in detail. The sensor’s applicability for the healthcare sector is demonstrated through breathing rate/pattern monitoring, skin moisture analysis, and neonatal care. To establish the wireless connectivity for real-time humidity monitoring a Raspberry Pi Pico-based system is used with a single-band 2.4 GHz wireless interface (802.11n, Infineon CYW43439). Further, the bending tests at different angles are performed to demonstrate the sensor’s potential efficacy for wearable sensing technologies. Moreover, the soil burial degradation test is also performed to study the biodegradability analysis of the used textiles. The originality of this work lies in proposing a simple, eco-friendly, and scalable approach with multinode wireless connectivity that offers a stable sensing performance toward a wide humidity range suitable for healthcare applications and opens a new pathway for sustainable and green electronics via mitigating the electronic waste (e-waste) problems.

## 2 Experimental Section

### 2.1 Fabrication of the PEDOT:PSS-Based Humidity Sensor

**Figure 1:**
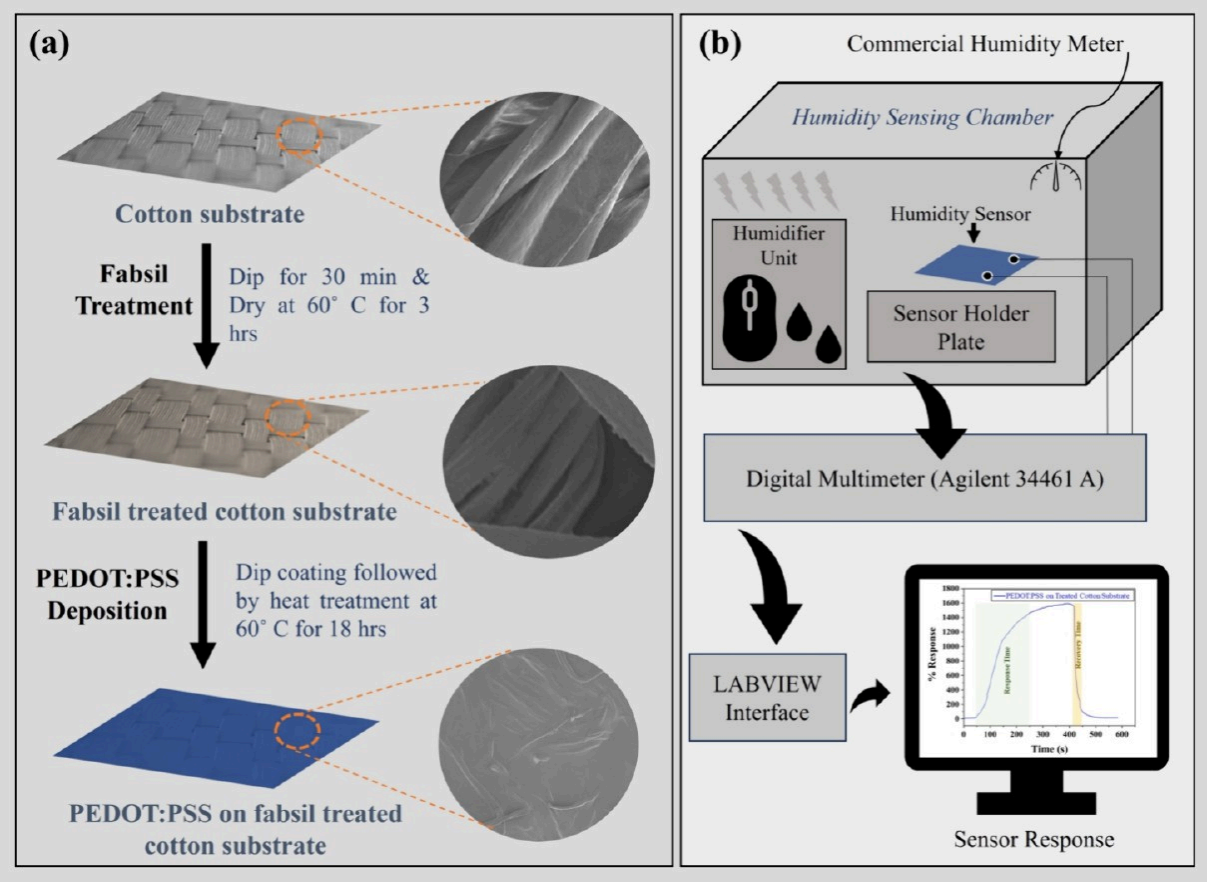
Schematic illustration of (a) sensor fabrication steps and (b) humidity sensing setup.

Commercially available cotton (textile) was used as substrate material, fabsil universal protector (commercially available) was used for textile treatment, and PEDOT:PSS (PH 1000, Ossila) was used as the active layer material. Figure 1a displays a schematic illustration of the sensor fabrication steps. The cotton substrate (dimensions ∼1 × 1 cm^2^) was treated with a fabsil protector (a silicone-based waterproof fabric protector) to make it double-face-hydrophobized cotton by dipping the substrate into fabsil solution (∼10 mL taken in a Petri dish) for 30 min. The cotton fabric was hanged for 5 min to remove the excess fabsil followed by a drying process carried out at 60 °C for 3 h. As treated and untreated (pristine) cotton substrates were subjected to PEDOT:PSS active layer deposition. The active/sensing layer was deposited using the dip coating method, i.e., dipping both the substrates into PEDOT:PSS solution for 15 min (based on trial-and-error experiments). The as-deposited layers were further processed by providing a heat treatment at 60 °C for 18 h to prepare PEDOT:PSS/pristine cotton sensor and PEDOT:PSS/fabsil treated cotton sensor.

### 2.2 Measurements and Humidity Sensing Setup

Fourier transform infrared spectroscopy (FTIR) analysis of the pristine cotton, fabsil-treated cotton, and PEDOT:PSS/fabsil-treated cotton samples was carried out using an FTIR spectrometer (Jasco FTIR 4100). Scanning electron microscopy (FEI Nova) and X-ray diffraction (XRD P’Analytical X‘Pert with Cu Kα (λ = 1.541 Å)) were used to study the structural and morphological characteristics of the deposited layer. Contact angle measurement was carried out using the sessile drop method to examine the hydrophilicity/hydrophobicity of the fabsil-treated and untreated cotton samples.

A schematic illustration of the humidity sensing setup is shown in Figure 1b. An in-house developed airtight acrylic sheet-based sensing chamber (dimensions: 50 cm × 40 cm × 45 cm) was used to evaluate the humidity sensing performance of the fabricated sensors. The sensors were placed individually on the sensor holder adjacent to a commercial humidity meter (ATP—Humidity and Temperature Meter DT-625 with accuracy of ±2%RH), which was used for calibration and observing humidity levels inside the chamber. To generate and precisely control the humidity levels, the humidifier unit (PureMate PM 908 Digital Ultrasonic Cool Mist Humidifier) was also placed inside the sensing chamber. The dehumidification process was accomplished by releasing the top panel and purging the fresh air inside the chamber. Moreover, the change in resistance behavior of the developed sensor was determined using the digital multimeter (Agilent 34461A 61/2 Digit Multimeter) and logged using the LabVIEW interface as shown in Figure 1b. The % response value is calculated using the following equation:
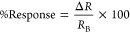
1where Δ*R* = *R*_A_ – *R*_B_; *R*_A_ is the resistance at a specified humidity level; *R*_B_ is the baseline resistance obtained at ∼25% RH.

## 3 Results and Discussion

### 3.1 Material Characterization

**Figure 2:**
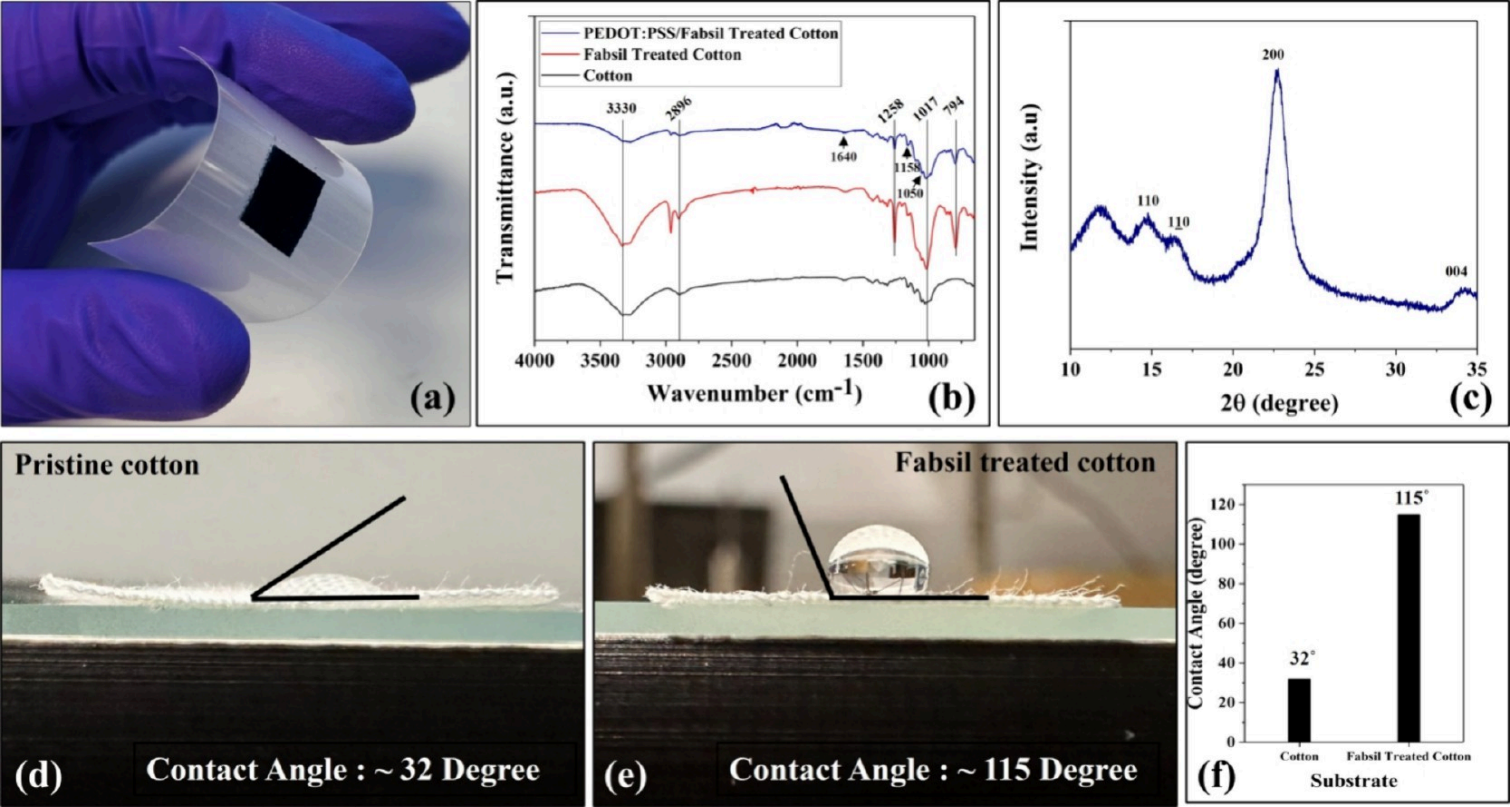
(a) Fabricated humidity sensor using PEDOT:PSS on fabsil-treated cotton attached on a sheet. (b) FTIR spectra of cotton, fabsil-treated cotton, and PEDOT:PSS layer on fabsil treated cotton. (c) X-ray diffraction of the PEDOT:PSS layer on the fabsil-treated cotton substrate. (d–f) Contact angle measurements of pristine and fabsil-treated cotton substrates.

The fabricated humidity sensor using PEDOT:PSS on fabsil-treated cotton substrate attached on a sheet is presented in Figure 2a. To determine the different functional groups of pristine cotton, fabsil-treated cotton, and PEDOT:PSS layer on fabsil-treated cotton, FTIR measurements were carried out, and the obtained results are shown in Figure 2b. Pristine and fabsil-treated cotton samples demonstrate distinct peaks associated with cellulose structure at 3330, 2896, and 1017 cm^–1^, which are ascribed to −OH, −CH, and C–C stretching bands, respectively.^38−41^ The fabsil-treated cotton sample displays additional stretching vibrations peaks at 1258 and 794 cm^–1^. The FTIR spectra of fabsil-treated cotton before and after PEDOT:PSS deposition exhibit almost similar peaks with minor shifts and reduced stretching bands, indicating the agglomeration tendency of PEDOT domains within the treated cotton substrate.^42^ Further, after the PEDOT:PSS layer deposition, the broad peak observed at 3330 cm^–1^ is reduced significantly, which is ascribed to the cross-linking reactions between the functionalized group of PEDOT:PSS and −OH groups of cellulose.^43^ The peaks observed at 1640 and 1158 cm^–1^ are assigned to C=C bond from the aromatic ring and symmetrical vibration (of SO_3_H) in PSS.^44^ The peak exhibited at 1050 cm^–1^ is attributed to the S–phenyl bond in PSS.^38^

To further investigate the structural synergies between sensing material PEDOT:PSS and the fabsil-treated cotton (textile) substrate, X-ray diffraction analysis was performed and the obtained XRD pattern is shown in Figure 2c. The obtained pattern displayed characteristic peaks with distinctive reflection planes at 2θ = 14.8° (110), 2θ = 16.2° (110), 2θ = 22.8° (200), and 2θ = 34.3° (004). The observed diffraction pattern is in good agreement with the background studies,^38,41,45^ indicating the successful incorporation of PEDOT:PSS within the cellulose matrix of the fabsil-treated cotton strands as required for sensor development. Furthermore, contact angle measurements were performed to understand the impact of fabsil treatment on the water absorbing property of the cotton substrate, as shown in Figure 2d–f. The contact angle for pristine cotton (as shown in Figure 2d) was measured as ∼32°, which indicates a super hydrophilic behavior of the cotton substrate.^46^ Further, the contact angle for the treated cotton substrate (as shown in Figure 2e) was found to be ∼115°, suggesting an enriched hydrophobicity post fabsil treatment. A comparative analysis of contact angle measurement is presented in Figure 2f. Along with this, microscopic images of PEDOT:PSS-coated layers were also acquired to display the successful deposition of PEDOT:PSS on treated and untreated cotton substrates, as shown in Figure S1a–d.

**Figure 3:**
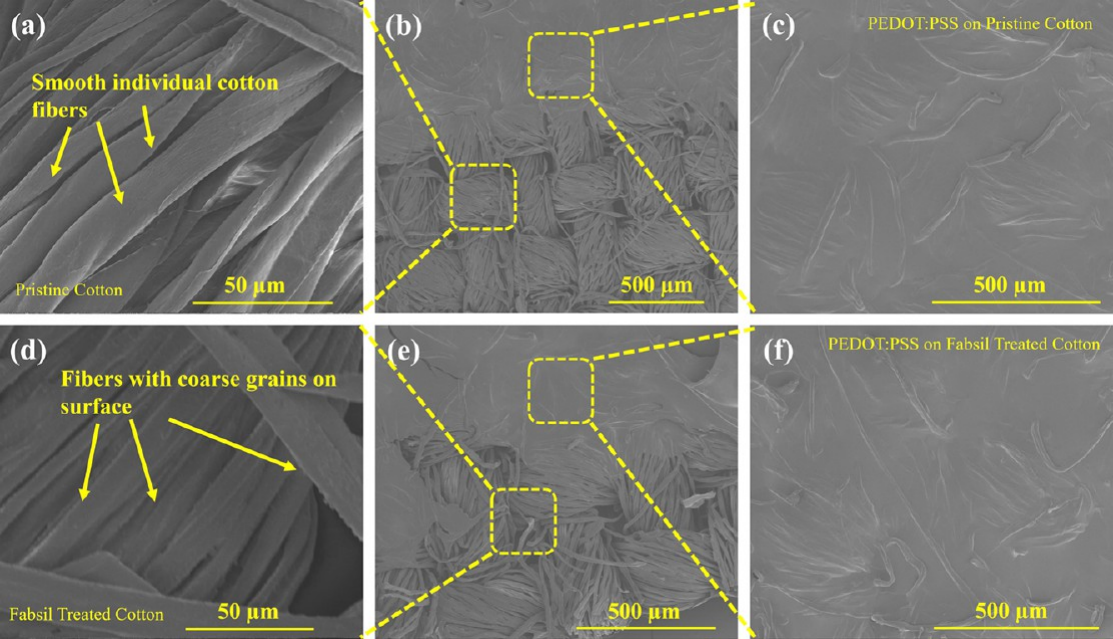
Morphological characteristics of pristine and fabsil-treated cotton. SEM images demonstrating (a) pristine cotton at 50 μm scale, (b) interface between the pristine cotton and the PEDOT:PSS/pristine cotton, (c) PEDOT:PSS deposited on pristine cotton at 500 μm scale, (d) Fabsil-treated cotton at 50 μm scale, (e) interface between the fabsil-treated cotton and the PEDOT:PSS/fabsil treated cotton, and (f) PEDOT:PSS deposited on fabsil-treated cotton at 500 μm scale.

Moreover, SEM analysis was performed to investigate the morphological characteristics of the pristine and fabsil-treated cotton samples. The images pre and post PEDOT:PSS deposition are shown in Figure 3a–f. The pristine cotton/textile samples show smooth surfaces, without noticeable particles on the textile fiber surfaces, as evident from Figure 3a. Successful deposition of the PEDOT:PSS sensing layer on the pristine cotton substrate is displayed in Figure 3b,c, which also shows the interface between the sensing layer and the substrate, whereas the fabsil-treated cotton sample exhibits coarse surfaces, having noticeable particles on the individual fiber surfaces as evident from Figure 3d. Successful deposition of the PEDOT:PSS sensing layer on the fabsil-treated cotton substrate is displayed in Figure 3e,f, which also represents the interface between PEDOT:PSS and the fabsil-treated cotton. The PEDOT: PSS-based sensing layer is observed along with filling the space between the individual cotton fibers, as shown in Figure 3f.

### 3.2 Humidity Sensing Properties

**Figure 4:**
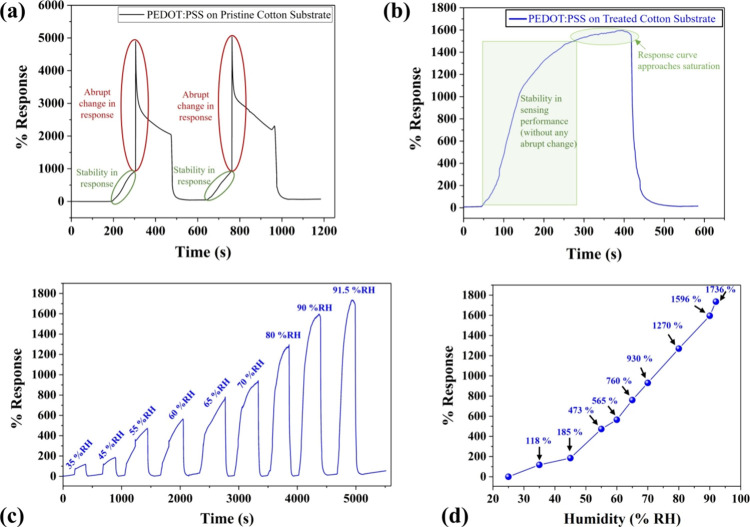
(a) Humidity sensing analysis of PEDOT:PSS/pristine cotton-based sensor in the range of 25–90% RH. Humidity sensing analysis of PEDOT:PSS/treated cotton-based sensor (b) in the range of 25–90% RH, (c) sensing analysis at intermediate RH levels (within 25–91.5% RH), and (d) % response vs % RH graph.

PEDOT:PSS-based sensors developed on pristine cotton (untreated textile) and fabsil-treated cotton substrates were tested within humidity of 25–90% RH. The obtained % responses based on the change in resistance signal are plotted in Figure 4a,b. The % response of the PEDOT:PSS/pristine cotton sensor demonstrates a linearly increasing trend (stability in response) until humidity reaches up to ∼70% RH, and beyond that, an abrupt change in response is observed as shown in Figure 4a. The observed trend in sensing response is further confirmed by repeating the sensing cycle. This abrupt change in the sensing response after a certain humidity level could be ascribed to moisture absorption in the cotton substrate due to its porous structures and super hydrophilic behavior^46^ (well supported by 32° contact angle), hence causing instability in the sensing response. Also, the impact of the moisture absorbance on the sensor’s lifetime is inevitable and may pose errors in the sensing response.^47^ However, it is desirable that a high-performance humidity sensor should have stable and linear/nearly linear response over the desired/broad RH range^1^ as stability and linearity of response to humidity is imperative for practical applicability.^27,48^ Therefore, the obtained results indicate the limited usage of the developed PEDOT:PSS/pristine cotton sensor for long-run and broad-range humidity sensing applications.

To overcome the stated limitation of the PEDOT:PSS/pristine cotton sensors, the cotton substrate was modified by implementing fabsil treatment to generate double-face-hydrophobized cotton, as discussed in the experimental section. The PEDOT:PSS/treated cotton-based sensor was also investigated within the humidity range 25–90% RH, and the results are shown in Figure 4b. The obtained sensing graph indicates the stability in sensing performance (without any abrupt change) as the response curve approaches saturation after reaching the maximum exposed humidity, i.e., 90% RH. This could be ascribed to enriched hydrophobicity (supported by 115° contact angle) of the treated cotton substrate, and hence, this diminished moisture absorption property of the treated cotton substrate led to a stable humidity sensing response.^49^ Owing to stable sensing performance, the PEDOT:PSS/treated cotton-based sensor was further explored for detailed characterizations and application considerations. Henceforth, this sensor is termed a “humidity sensor”, unless otherwise indicated.

The sensing characteristics of the developed humidity sensor were also investigated at different intermediate RH levels (within 25–91.5% RH), and the results are shown in Figure 4c. The sensor was exposed to humid air having a humidity of 35% RH, 45% RH, 55% RH, 60% RH, 65% RH, 70% RH, 80% RH, 90% RH, and 91.5% RH, and the % responses are 118, 185, 473, 565, 760, 930, 1270, 1596, and 1736%, respectively. The % response vs % RH is plotted in Figure 4d. The linearity coefficient (Adj. *R*-square) value is 0.95035, revealing a nearly linear humidity sensing response within the considered RH range. Other important parameters include response time (change in % response up to 90% of the equilibrium value)^1,50,51^ and recovery time (change in % response up to 10% of the equilibrium value)^1,50,51^ of the sensor, which have been found to be ∼200 and ∼30 s, respectively, as shown in Figure S2.

### 3.3 Repeatability and Reproducibility Analysis

**Figure 5:**
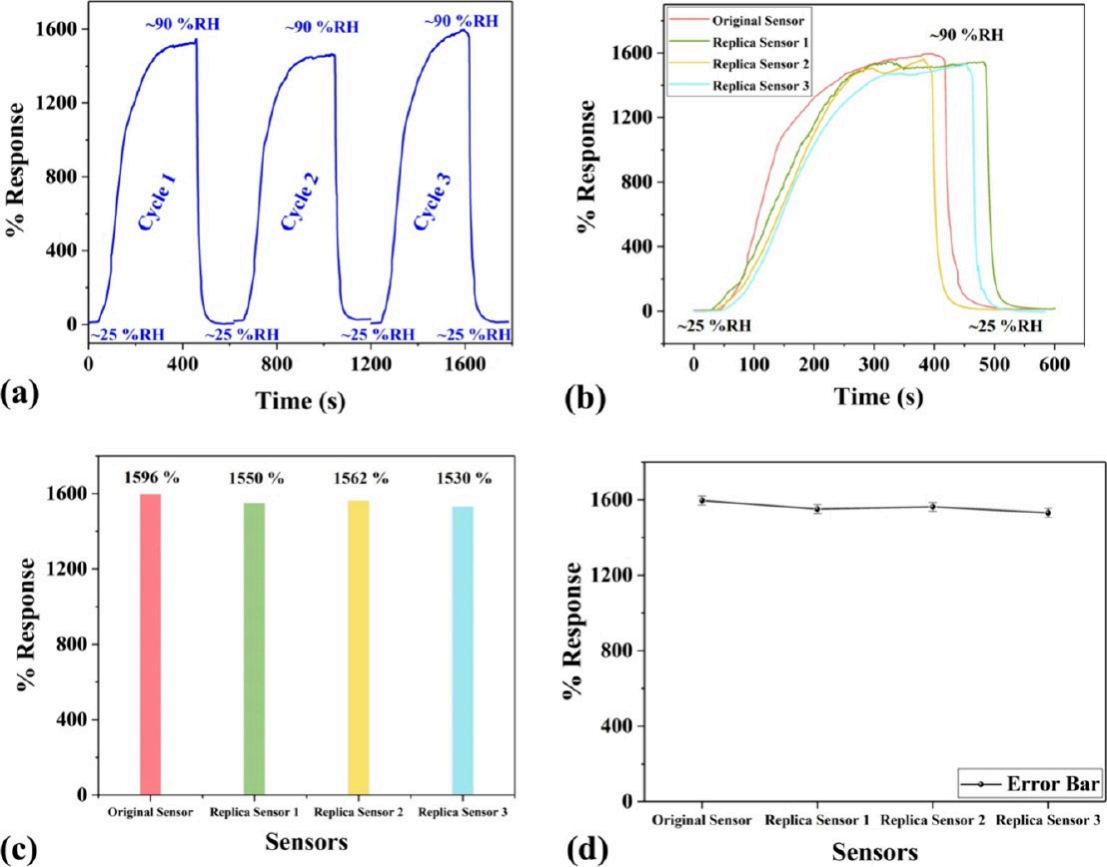
(a) Three cyclic repeatability analysis in the range of 25–90% RH. (b) Reproducibility analysis of the original and three replica sensors. (c) Comparative analysis of % responses between the original and the replica sensors and (d) error presentation for reproducibility analysis.

The humidity sensor was further examined for repeatability and reproducibility. A three cyclic repeatability analysis was performed by running three individual humidity tests in the range of 25–90% RH. The attained results, as shown in Figure 5a, signify repeatable humidity sensing characteristics. Furthermore, three replica sensors (termed replica sensors 1, 2, and 3) were made using the same fabrication method as discussed in the experimental section for the reproducibility analysis. The replica sensors were also exposed to humidity of 25–90% RH. The results for replica sensors 1, 2, and 3 are plotted and compared with the original humidity sensor in Figure 5b. The comparative performance (in % response) of the replicas with the original sensor is denoted in Figure 5c. The responses for the replica sensors are perceived well in accordance with the humidity/original sensor with insignificant variations. The response and recovery times of the replica sensors are also found to be in good accordance with the original sensor. A comparative analysis of the response and recovery times of the original sensor with the replica sensors is displayed in Figure S3. Further, the error bar for the three replicas along with the original sensor is represented in Figure 5d. The margin of error with a confidence level of 99% is also calculated and found to be ±1.98%. In addition, the aging effect (i.e., stability of sensor with time^52^) on the sensing performance was also evaluated by monitoring and comparing its performance at Week 1 and Week 21 in Figure S4. The result depicts a highly stable response of the sensors having only a small drop (∼0.91%/%RH) in sensing performance. These results clearly indicate the high performance of the developed humidity sensors and their applicability for various application areas including wearable healthcare devices.

### 3.4 Sensor Applications for Healthcare

Breathing or respiration rate monitoring is vital for evaluating human health as abnormal breathing is related to many health problems in human beings.^27^ Several discomforts and illnesses, such as bronchitis, heart diseases, pneumonia, chronic obstructive pulmonary disease (COPD), sleep apnea syndrome (SAS), asthma, etc. could lead to changes in breathing rate and depth.^27−30^ Any breathing rate more than 24 breaths per minute (bpm) for a prolonged duration (e.g., several hours) is considered a health risk or underlying health condition^53^ because the usual breathing rate (at rest condition) of a healthy human being is roughly 12–20 bpm.^29^ If the breathing rate is greater than 27 bpm, it is considered a vital sign for cardiac/cardiopulmonary arrest in health centers.^54^ Therefore, breathing rate monitoring is a useful approach in healthcare and can help in several healthcare assessments.

**Figure 6:**
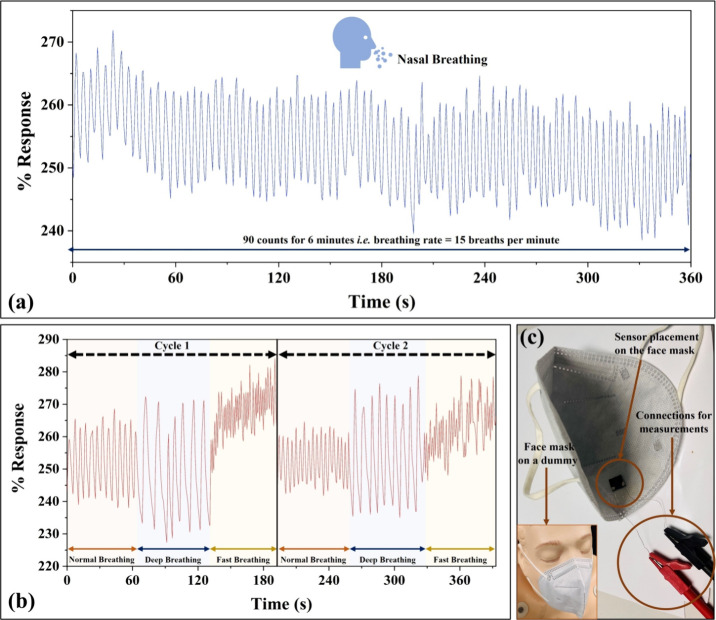
(a) % Response vs time graph for continuous nasal breathing rate monitoring for 6 min. (b) Sensor’s response against different breathing patterns such as 2-cycles of normal, deep, and fast breathing. (c) Humidity sensor placement on the face mask for breathing rate monitoring.

Humidity sensing using a chemiresistive sensor is a promising route to determine a relation between electrical response (change in resistance) and breathing rate. The developed humidity sensor was explored for monitoring the human breathing rate. Different breathing patterns have been tested and the results are shown in Figure 6a–c. The nasal breathing rate/patterns monitoring was performed by attaching the sensor to a face mask, as shown in Figure 6c. The breathing rate of an adult (at rest condition) was evaluated by monitoring the % response of the sensor with exhale/inhale cycles, as shown in Figure 6a. The nasal breathing rate was continuously monitored for 6 min, and the recorded number of clearly distinguishable peaks (90 peaks observed corresponding to exhale/inhale cycles) indicates a breathing rate of 15 bpm. Further, the sensor was also investigated against different breathing patterns such as 2-cycles of normal, deep, and fast breathing, as shown in Figure 6b. The results show that the sensor can easily distinguish among different breathing patterns in addition to monitoring the breathing rate, indicating the sensor’s suitability toward breathing rate/patterns monitoring and opening possibilities in healthcare applications. Moreover, the flexibility and breathability^55^ of the textiles seem highly advantageous for further enhancing their wearability in respiration rate monitoring as the sensor can be directly knitted with the face mask.

**Figure 7:**
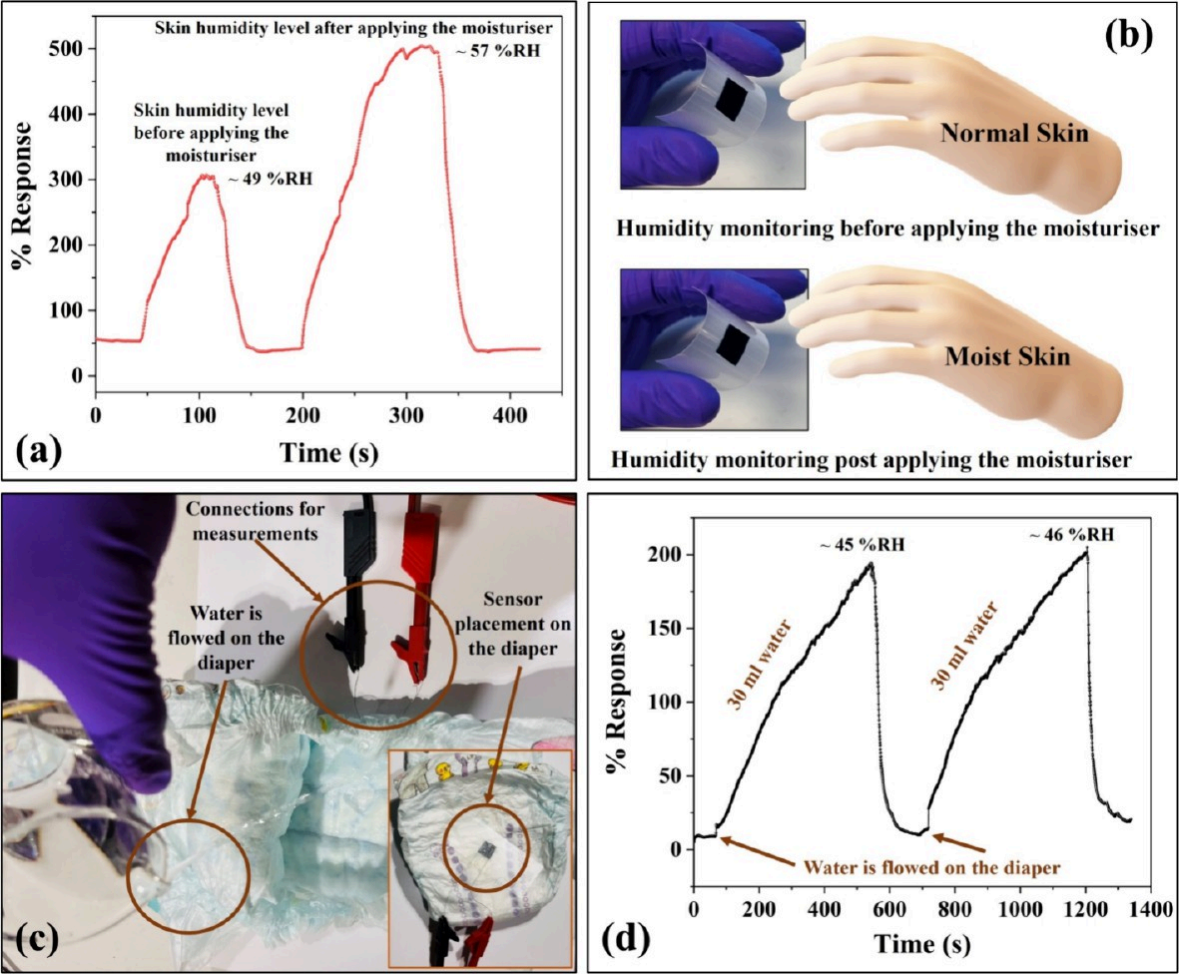
(a) % Response vs time when monitoring skin humidity before and after applying skin moisturizer. (b) Schematic representation for skin moisture monitoring pre and post moisturized conditions. (c) Experimental setup for neonatal care application and (d) two-cyclic analysis for diaper wetting process: % response vs time.

Furthermore, skin moisture monitoring is another application area for the developed sensor. Unbalanced (either too low or elevated) skin humidity levels can lead to several skin conditions such as fungal infections, dry skin, eczema, allergies, etc.^5,34,56^ Also, skin moisture monitoring can provide insights into the physiological state, wound healing monitoring, and dehydration-related diseases/conditions (particularly for sportspersons).^56,57^ Therefore, a humidity sensor can be employed to take precautionary measures based on the skin humidity levels. We tested the developed sensors by measuring the skin humidity levels pre (normal skin) and post (moist skin) by applying a commercial moisturizer, and the results are shown in Figure 7a,b. Based on the % responses observed for both cases, as shown in Figure 7a, the skin humidity levels are found to be ∼49% RH and ∼57% RH. The sensor results for skin humidity levels were found to be in good agreement (with an accuracy ±2%RH) with the levels monitored using the commercial humidity meter.

Another potential application of the developed humidity sensor was studied in neonatal care (diaper wetting) applications. Prolonged exposure to wet diapers or infrequent change of diapers may lead to several conditions for the infants such as skin irritation and painful rashes.^35,58^ Therefore, a diaper with a sensor attached to its inner surface, as shown in Figure 7c, was investigated for providing useful information about humidity level. To simulate the baby's urinal discharge, 30 mL of water was discharged on the diaper to monitor the change in % response (corresponding to a change in the humidity level) via two-cyclic repeatability analysis, as shown in Figure 7d. The humidity levels in relation to % responses were observed as ∼45% RH and ∼46% RH, respectively, for cycle 1 and cycle 2. The observed responses of the humidity sensor toward a diaper-wetting process indicate its excellent monitoring performance. Further, the observed humidity values were found to be in agreement (with an accuracy of ±2% RH) with the humidity levels monitored using a commercial humidity meter. Therefore, the obtained sensing performance of the developed sensor suggests its suitability for neonatal care applications. The sensor performance observed toward multiple healthcare applications opens up avenues for its possible applicability in practical scenarios.

### 3.5 Multinode Wireless Connectivity

**Figure 8:**
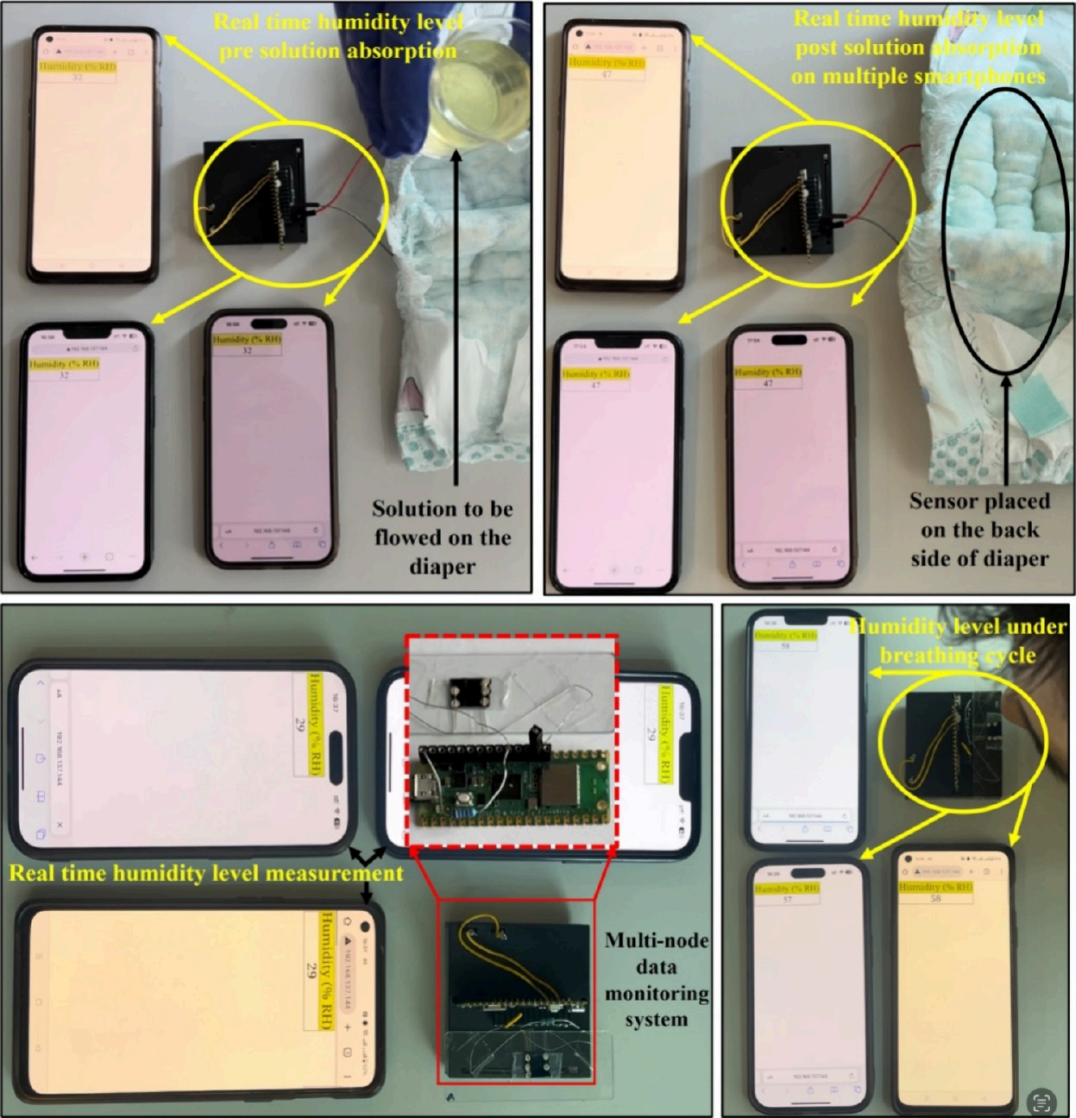
Real-time multinode humidity sensor data monitoring on a multiple smartphones (a, b) humidity levels pre and post simulated urine solution absorption on diaper and (c, d) humidity level measurements under breathing cycle (inhale and exhale).

Real-time multinode data monitoring and control is vital for the effective utilization of the sensors in healthcare devices. Herein, a Raspberry Pi Pico-based system is used to read the real-time analog data from the fabricated humidity sensor. The system uses an RP2040 microcontroller with a Dual-core Arm Cortex M0+ processor and a single-band 2.4 GHz wireless interface (802.11n, Infineon CYW43439), which can be programmed using MicroPython. The humidity sensor is connected in a voltage divider scheme with a balancing resistor between 5 V source voltage and ground, as shown in Figure S5a. As the resistance of the sensor changes (due to the influx of moisture on its active surface), the voltage drop across the sensor also varies proportionately which is logged by the analog-to-digital converter (ADC) pin with respect to time. This variation in voltage is used to calculate the level of humidity in the immediate neighborhood of the sensor. The analog voltage data are first recorded by an ADC pin on the Raspberry Pi and converted to corresponding humidity levels using the calibration values previously recorded from the fabricated humidity sensor. The data are then transmitted from the Pi (acting as a server) to clients using the Wi-Fi TCP/IP protocol. The humidity level can be accessed simultaneously by any general-purpose hyper-text transfer protocol browser on multiple devices that are connected to the same Wi-Fi as the Raspberry Pi as shown in Figure 8a–d, where the %RH values are displayed on the browsers of multiple smartphones, thus removing the necessity of designing a separate software application to read the sensor data. Moreover, the real-time breath count measurement is also realized and examined for different breathing patterns (normal, deep, and fast), as shown in Figure S5b. Furthermore, real-time multinode humidity sensor data monitoring on multiple mobile phones is demonstrated (Supplementary Videos S1 and S2) for neonatal care and breathing rate monitoring applications. For real-time monitoring, the sensing properties of the sensor are tested by replacing the 30 mL of water with simulated urine solution in a diaper wetting experiment as shown in Figure 8a,b and Supplementary Video S1. The humidity level in relation to % response is observed as ∼47%RH with simulated urine which is found to be well in accordance with the water (as humidity level in relation to % response is observed as ∼46%RH with water). Figure 8c,d and Supplementary Video S2 demonstrate the humidity level measurements under the breathing cycle (inhale and exhale) where the %RH increases from 29%RH under exposure to moisture from exhaled nasal breath.

### 3.6 Bending Tests

**Figure 9:**
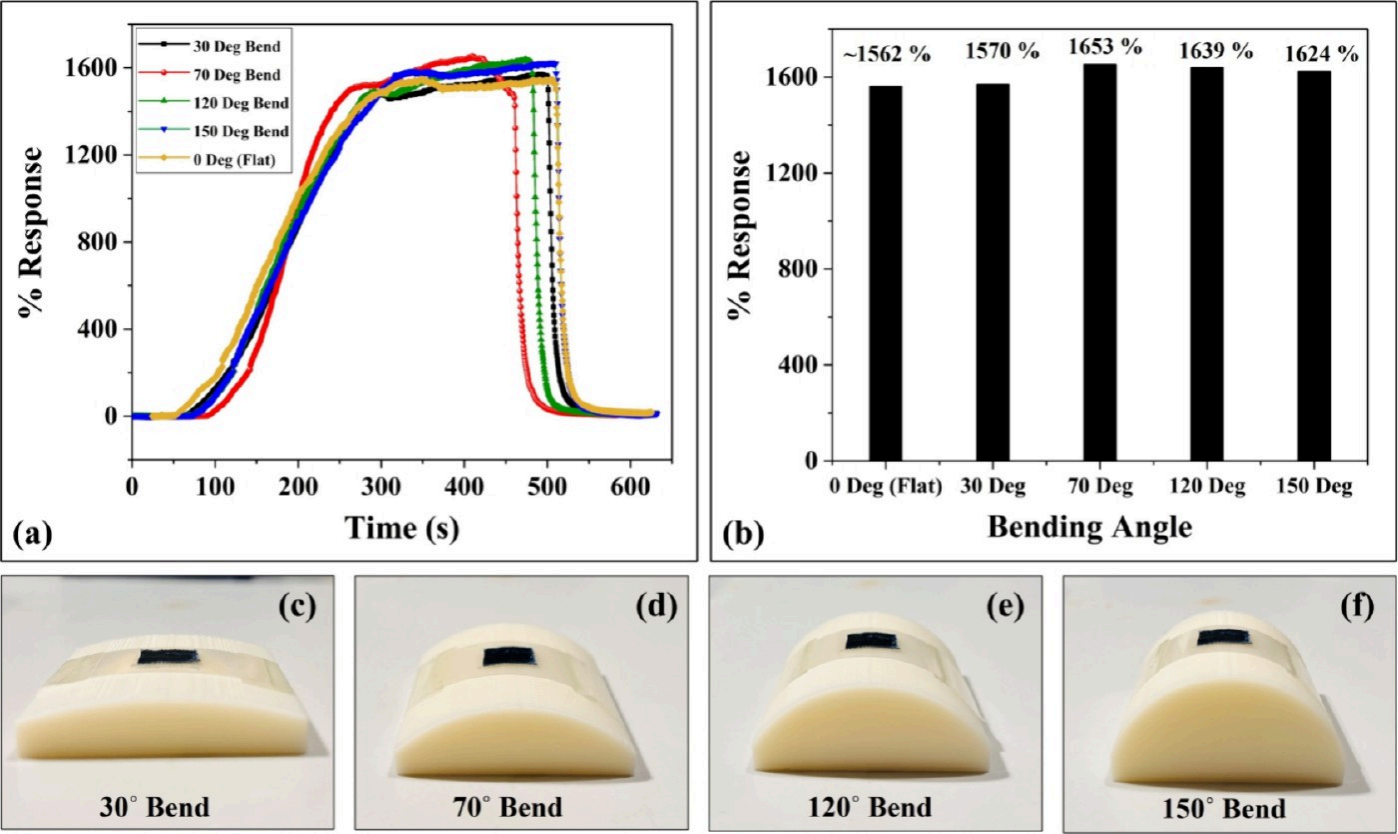
(a) % Response vs time graph for flat (0°), 30°, 70°, 120°, and 150° bending angles. (b) Comparative analysis graph of bending condition responses with the flat (0°) condition response. Bending setup used for (c) 30°, (d) 70°, (e) 120°, and (f) 150° bending conditions.

The bending test is vital and plays an important role in promoting the sensor’s applicability in wearable sensing technologies.^11^ We performed bending tests on one of the three replica humidity sensors, and the results are displayed in Figure 9a–f. The humidity sensing performance of the developed sensor was investigated under different angles such as 30°, 70°, 120°, and 150°. The bending setup used for different conditions is displayed in Figure 9c–f. The sensing performance under different bending angles was investigated individually in the RH range of 25–90% RH and compared with the flat or unbended (0°) condition and the sensing responses in different scenarios are shown in Figure 9a. The sensing responses are ∼1570, 1653, 1639, and 1624% for 30°, 70°, 120°, and 150° bending angles, respectively, whereas it is ∼1562% under the flat condition. The margin of error with a confidence level of 99% is determined and found to be ±2.64%. The results demonstrate excellent flexibility and stability of the sensors making them highly suitable for the potential wearable applications.

### 3.7 Biodegradability Analysis

**Figure 10:**
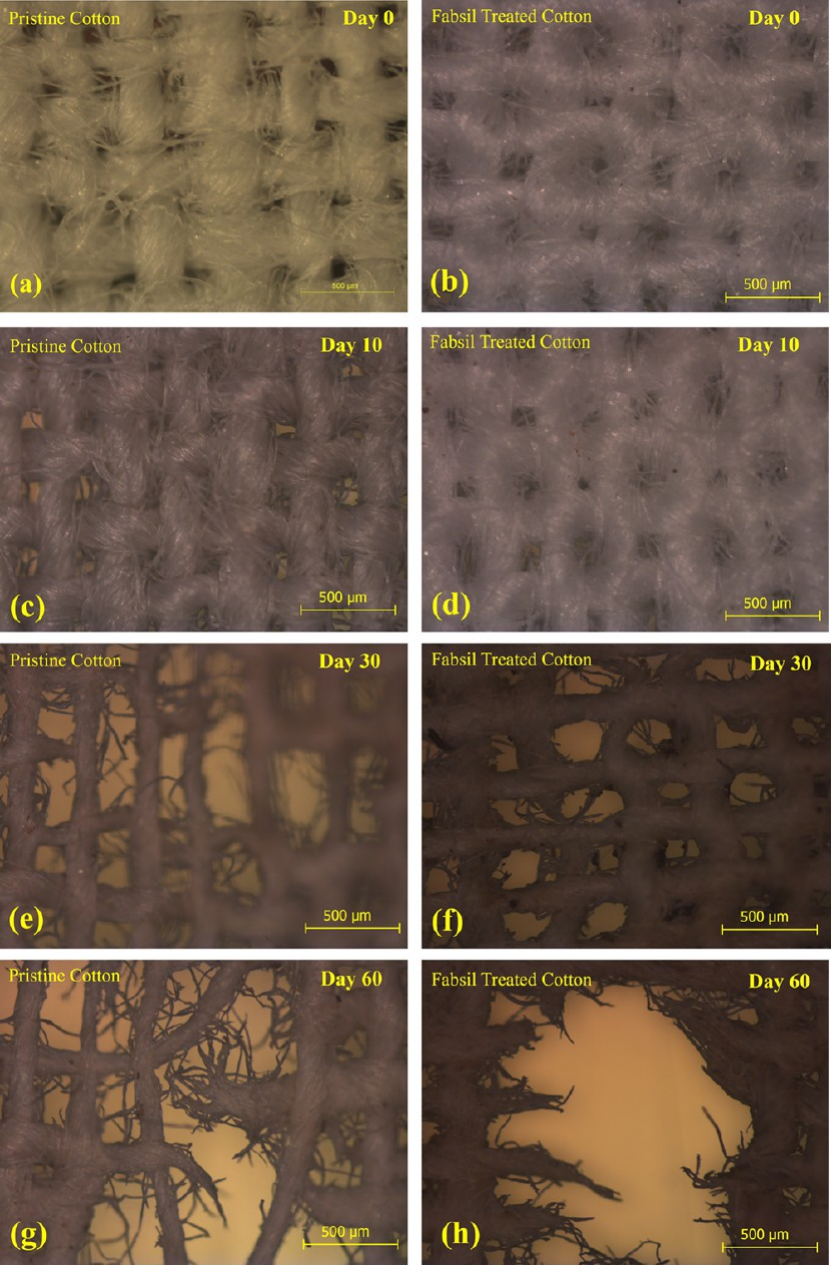
(a) Pristine and (b) fabsil-treated cotton samples at a 500 μm scale before being buried. Microscopic images (at 500 μm scale) of both samples depicting degradation after (c, d) 10 days, (e, f) 30 days and (g, h) 60 days of soil burial.

The soil burial degradation test was used to examine the bio-degradability of the pristine and fabsil-treated cotton (textile) substrates. Both the samples were buried inside multipurpose compost soil for a prolonged duration. The degradation or disintegration rate of both samples has been continuously monitored for 60 days, and results are displayed in Figure 10a–h. The buried samples retrieved from compost soil after different durations (10, 30, and 60 days) were thoroughly cleaned and imaged under a microscope at a scale of 500 μm. The buried samples retrieved after 10 days (Figure 10c,d) demonstrate insignificant degradation in both the samples as compared to the unburied ones (Figure 10a,b). Substantial degradation is observed in both buried samples when retrieved after 30 days, as shown in Figure 10e,f. Furthermore, more significant disintegration and degradation of the textile fibers/threads is observed in both samples after 60 days as shown in Figure 10g,h. The degradation of cellulose fabric in soil is attributed to the presence of fungi and cellulolytic bacteria. Such microorganisms secrete an enzyme that leads to the cleavage of glycosidic bonds or can hydrolyze the β-1,4-glycosidic linkages.^26^ The degradation of uncoated cotton fabric was faster compared to the fabsil-treated sample due to the time taken by moisture to remove the coating that allows the microorganisms to act on the cotton fabric. The obtained results indicate that even though the fabsil treatment of the cotton/textile sample was still susceptible to biodegradation, the environmentally friendly nature of the developed humidity sensors paves the way toward curtailing the e-waste issue.

### 3.8 Humidity Sensing Mechanism and Comparative Analysis

**Figure 11:**
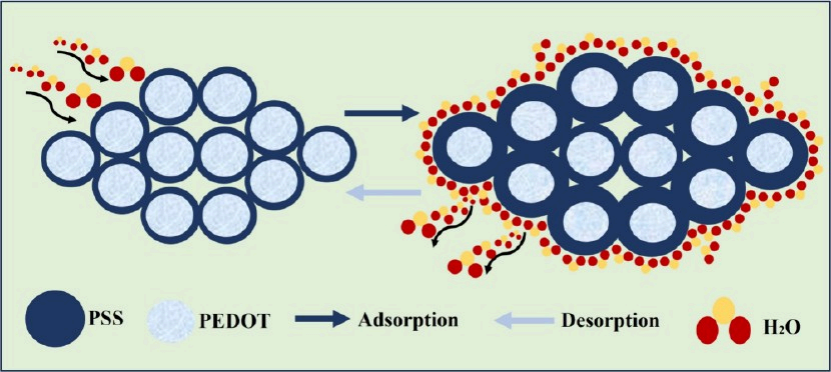
Schematic illustration of the humidity sensing mechanism.

The humidity sensing mechanism of the developed PEDOT:PSS-based sensor is illustrated in Figure 11. The hygroscopic nature of the PEDOT:PSS makes it a promising humidity-sensing material.^36,59^ The water adsorption and desorption phenomenon defines the humidity sensing mechanism of PEDOT: PSS-based sensors.^60,61^ PEDOT:PSS is a core–shell structure consisting of a conductive and hydrophobic PEDOT core and insulating and hydrophilic PSS shell.^62,63^ As the sensor is exposed to highly humid/moist conditions, the PSS shell layer seems to adsorb water molecules and expand, resulting in an increase in sensor resistance due to expansion in the distance between adjacent hydrophobic and conductive PEDOT enriched cores.^36,64^ This increased distance/barrier at the grain boundary limits the charge carrier hopping between the PEDOT:PSS cores.^65^ Similarly, the desorption process (low humidity) results in shrinkage of the PSS shell layer, and hence, the distance between adjacent conductive PEDOT enriched cores reduces and conductivity increases i.e., resistance decreases.^64^

Moreover, a comparative analysis of the present work is also presented with the background studies. The performance is comparatively assessed using various parameters considering potential wearable applicability and eco-friendly nature of the developed humidity sensor as illustrated in Table 1.

**Table 1:** Comparative Analysis of Humidity Sensing Performance of PEDOT:PSS/Fabsil-Treated Textile-Based Sensor with Resistive-type State-of-the-Art Works

material composition	substrate	humidity range (%RH)	sensitivity or response	degradability analysis	flexibility	ref
1D-nanoconfined PEDOT:PSS	PET substrate	0–13%	5.46%	no	yes	(64)
PEDOT:PSS-coated graphene–carbon layer	PVC substrate	25–90%	∼1%/%RH	no	yes	(66)
rGO/WS_2_	silicon	0–91.5%	∼0.18%/%RH	no	no	(67)
functionalized MWCNT/hydroxyethyl cellulose	PET film	20–80%	0.048/%RH	no	yes	(68)
rGO/poly(diallylimethyammonium chloride)	polyimide substrate	11–97%	8.69–37.41% i.e., 0.33/%RH	no	yes	(69)
graphene	silicon	1–96%	0.31%/%RH	no	no	(70)
MWCNTs	Kapton	10–90%	0.69%/%RH	no	yes	(71)
cellulose nanofiber/carbon black composite	polyethylene naphthalate	30–90%	120% i.e., 2%/%RH	no	yes	(72)
PEDOT:PSS	cotton (textile)	25–91.5%	26.1%/%RH	yes	yes	this work

Based on the comparative analysis, it can be found that the PEDOT:PSS/textile-based wearable sensor presented in this work exhibits an excellent and superior performance having high sensitivity (26.1%/%RH) for a wide humidity range (25–91.5%). Further, the biodegradability results presented here support innovative growth in sensing technology coupled with promoting a shift toward eco-friendly electronics.

## 4 Conclusions

In summary, a treated cotton (textile)-based eco-friendly humidity sensor suitable for wearable healthcare applications with real-time monitoring is reported. The pristine cotton sample was double-face-hydrophobized via fabsil treatment to obtain desired sensing characteristics. The PEDOT:PSS was used as the active layer material, deposited using the dip coating method for the presented resistive-type humidity sensor. The sensor was investigated for a wide humidity sensing range (25–91.5%RH), considering potential applicability of the sensor for wearable healthcare devices. The sensor has a nearly linear response with a linearity coefficient of value 0.95035 and high % response of 1736% at 91.5%RH. The humidity sensor shows excellent response toward breathing rate and pattern monitoring, skin moisture monitoring, and neonatal care applications. The multinode wireless communication is established using a Raspberry Pi Pico-based system for proposing a real-time humidity monitoring system for the healthcare sector. The bending test conducted under 30°, 70°, 120°, and 150° bending conditions demonstrated the excellent flexibility of the sensor, suggesting its suitability for wearable applications. The environmentally friendly nature of the textile was promoted by the obtained biodegradability results. Thus, the results imply that the developed humidity sensor can be employed in wearable healthcare devices via intelligent electronic interfaces along with contributing to a cleaner, sustainable, and greener environment.

## Supporting Information Available

Microscopic images of PEDOT:PSS-coated layers on pristine cotton and fabsil-treated cotton substrates at 500 and 200 μm scales; response and recovery time analysis; comparative analysis of the response and recovery times of the original sensor with the replica sensors; stability analysis by comparing the week first humidity sensing performance with the response obtained on week 21st; schematic illustration of the multinode wireless connectivity system, and real-time breath counts measurement (PDF)
Real-time multinode humidity monitoring on multiple mobile phones for neonatal care (MP4)
Real-time multinode humidity monitoring on multiple mobile phones for breathing rate monitoring applications (MP4)
