# Supplementary material for: Eco-Friendly Textile-Based Wearable Humidity Sensor with Multinode Wireless Connectivity for Healthcare Applications
Source: ACS Appl Bio Mater. 2024 Jul 4;7(7):4772–84. doi: 10.1021/acsabm.4c00593 (PMC11253092; doi:10.1021/acsabm.4c00593)
Supplement: Supplementary file 1 — mt4c00593_si_001.pdf [file mt4c00593_si_001.pdf]

Supporting Information

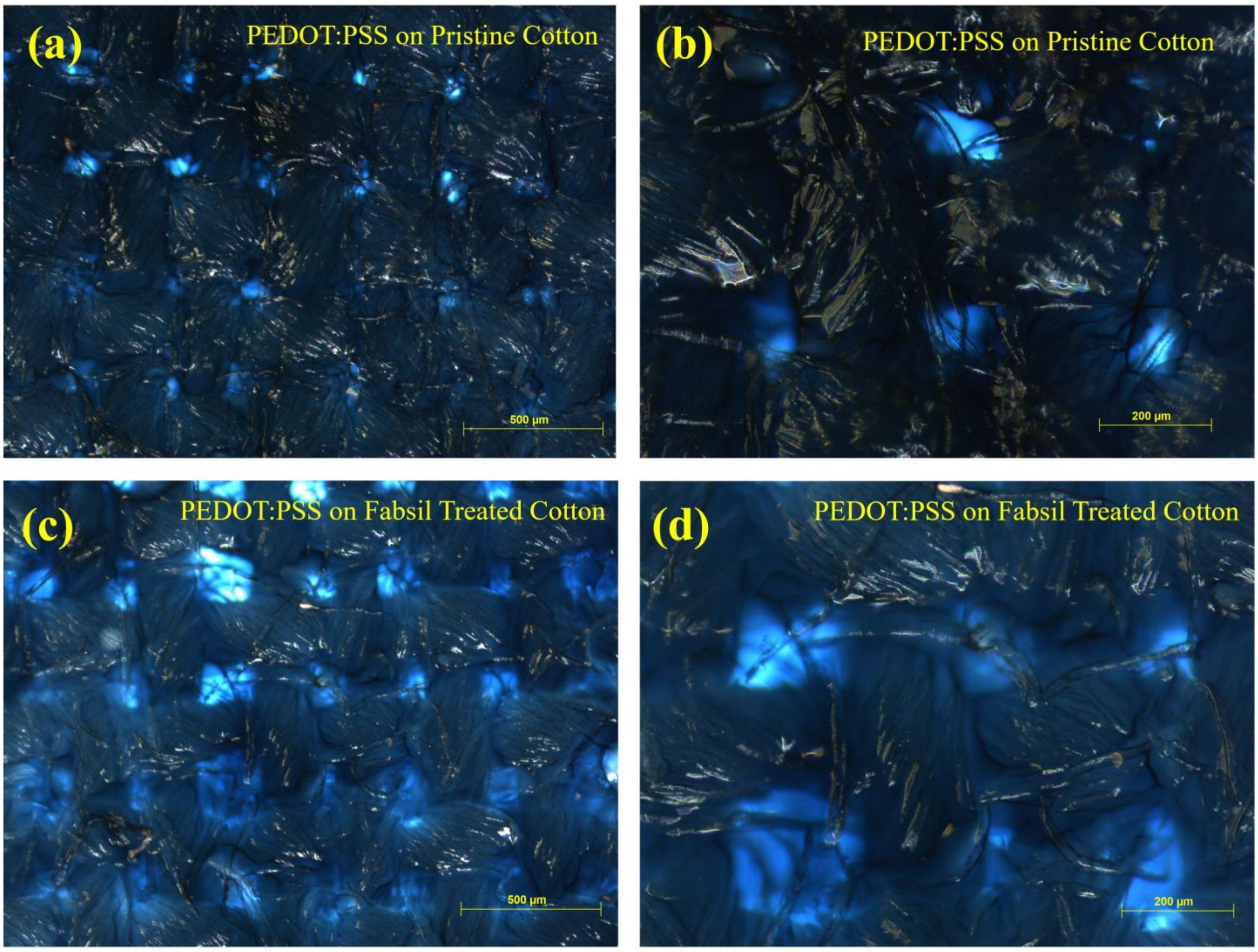

Figure S1. Microscopic images of PEDOT:PSS coated layers (a-b) pristine cotton and (c-d) fabsil treated cotton substrates at 500 µm and 200 µm scales.

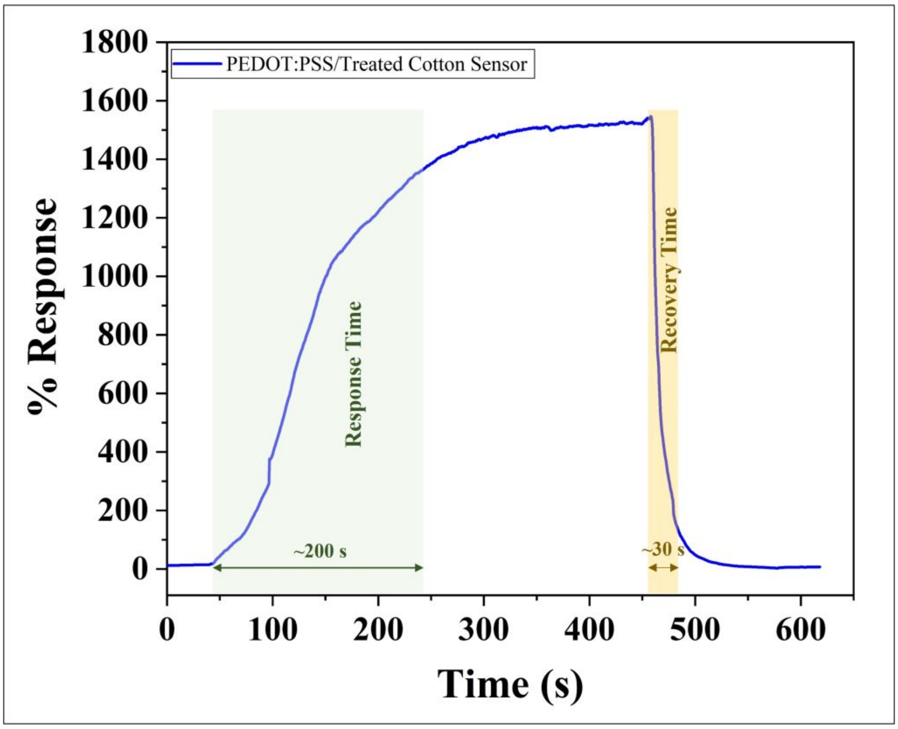

Figure S2. Response and recovery time analysis.

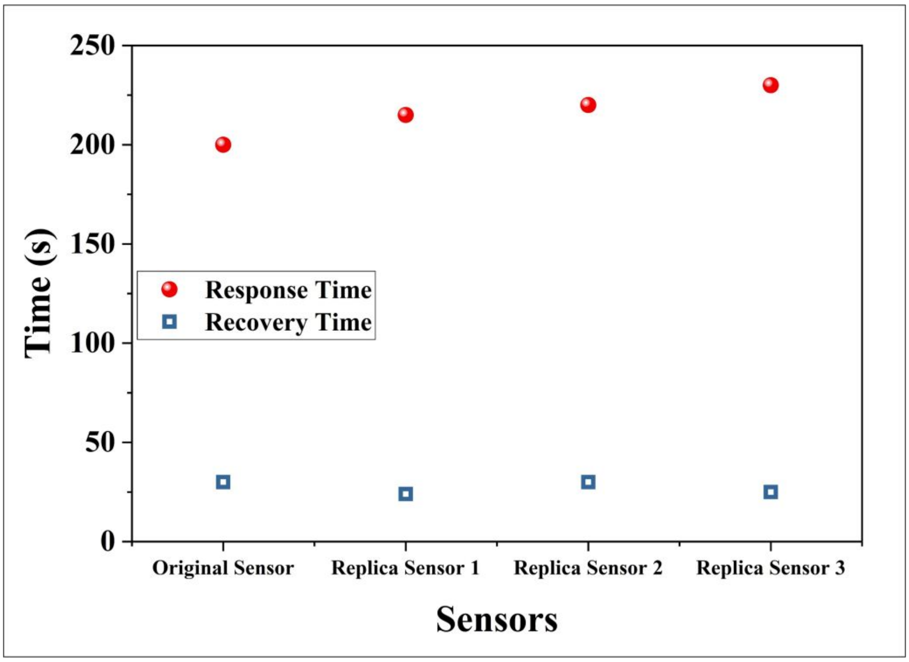

Figure S3. Comparative analysis of the response and recovery times of the original sensor with the replica sensors.

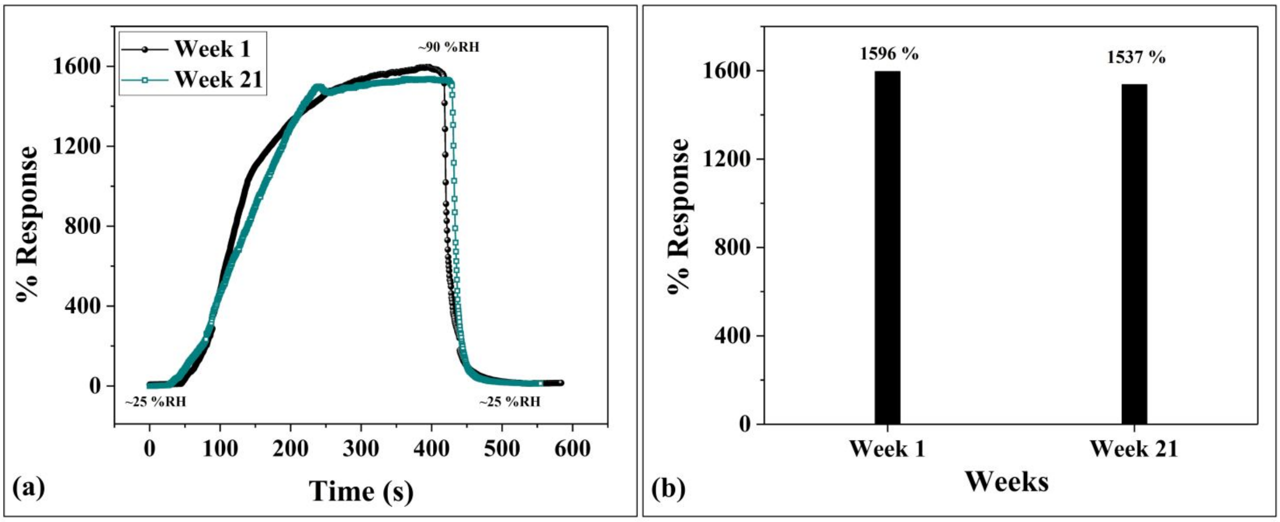

Figure S4. Stability analysis by comparing the week 1 humidity sensing performance with the response obtained on week 21.

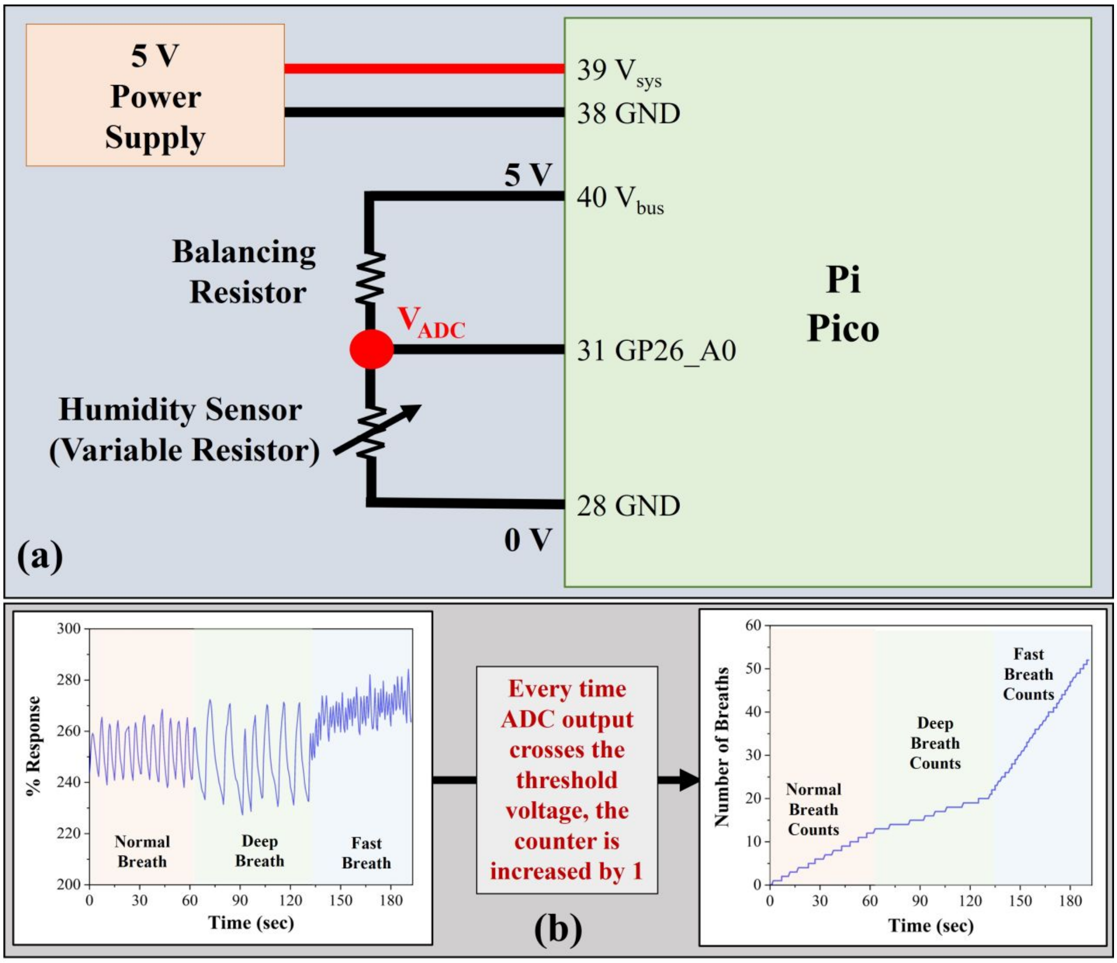

Figure S5. (a) Schematic illustration of the multi-node wireless connectivity system. (b) Real-time breath counts measurement.

Supplementary Video 1

This video presents the real-time multi-node humidity monitoring on mobile phones for neonatal care application.

Supplementary Video 2

This video presents the real-time multi-node humidity monitoring on mobile phones for breathing rate monitoring application.
